# P66Shc: A Pleiotropic Regulator of B Cell Trafficking and a Gatekeeper in Chronic Lymphocytic Leukemia

**DOI:** 10.3390/cancers12041006

**Published:** 2020-04-19

**Authors:** Laura Patrussi, Nagaja Capitani, Cosima T. Baldari

**Affiliations:** Department of Life Sciences, University of Siena, via Aldo Moro 2, 53100 Siena, Italy; capitani2@unisi.it

**Keywords:** CLL, p66Shc, receptor recycling, BCL2, apoptosis, homing receptors, lymphocyte trafficking

## Abstract

Neoplastic B cells from chronic lymphocytic leukemia patients (CLL) have a profound deficiency in the expression of p66Shc, an adaptor protein with pro-apoptotic and pro-oxidant activities. This defect results in leukemic B cell resistance to apoptosis and additionally impinges on the balance between chemokine receptors that control B cell homing to secondary lymphoid organs and the sphingosine phosphate receptor S1PR1 that controls their egress therefrom, thereby favoring leukemic B cell accumulation in the pro-survival lymphoid niche. Ablation of the gene encoding p66Shc in the Eµ-TCL1 mouse model of human CLL enhances leukemogenesis and promotes leukemic cell invasiveness in both nodal and extranodal organs, providing in vivo evidence of the pathogenic role of the p66Shc defect in CLL pathogenesis. Here we present an overview of the functions of p66Shc in B lymphocytes, with a specific focus on the multiple mechanisms exploited by p66Shc to control B cell trafficking and the abnormalities in this process caused by p66Shc deficiency in CLL.

## 1. Introduction

Among haematological malignancies, chronic lymphocytic leukemia (CLL) is a paradigm of how dysfunctions in the apoptotic machinery can affect disease onset and development. The hallmark of CLL is the accumulation of a peculiar monoclonal population of neoplastic CD5^+^CD19^+^ B cells in peripheral blood, bone marrow and lymphoid organs [1]. While a small fraction of CLL cells undergoes active proliferation in lymphoid niches termed “pseudofollicles” through their cross-talk with accessory cells [2,3], the majority of CLL cells accumulate as the result of apoptosis defects. Several factors contribute to the intrinsic predisposition of CLL B cells to arrest in the G0/G1 phases of the cell cycle without undergoing apoptosis [4]. These include genomic alterations such as 13q, 11q or 17p deletions or chromosome 12 trisomy [5], point mutations that lead to dysfunctional tumor suppressors [5] or hyperactivated mitogenic molecules [4,6], and epigenetic mechanisms leading to the overexpression of pro-survival molecules [7,8,9]. In addition to these intrinsic factors, several reports have recently highlighted the complex interplay between CLL B cells and the lymphoid microenvironment as a source of extrinsically-derived factors which make leukemic cells more prone to circumvent apoptosis [1,10]. This is further exacerbated by the abnormally high expression of chemokine receptors on the surface of CLL B cells, which promotes their trafficking and favors their retention in the pro-survival stromal niche [11,12].

A master regulator that coordinates intrinsic and extrinsic factors to protect CLL B cells from apoptosis and prolong their lifespan is still missing, although the adaptor protein p66Shc is a good candidate. Starting with the finding that p66Shc expression is profoundly impaired in CLL B cells [13], and based on its ability to negatively regulate survival signaling and promote oxidative stress-dependent apoptosis [14,15], over the past few years we have investigated the link between p66Shc expression, B cell apoptosis and B cell trafficking in CLL. In this review, we will provide an overview of the pro-apoptotic, anti-mitogenic and anti-chemotactic activities of p66Shc in the context of the pathogenesis of CLL.

## 2. The Multifaceted Role of Shc Proteins in Lymphocytes

### 2.1. The Shc Family of Adaptor Proteins 

**Figure 1:**
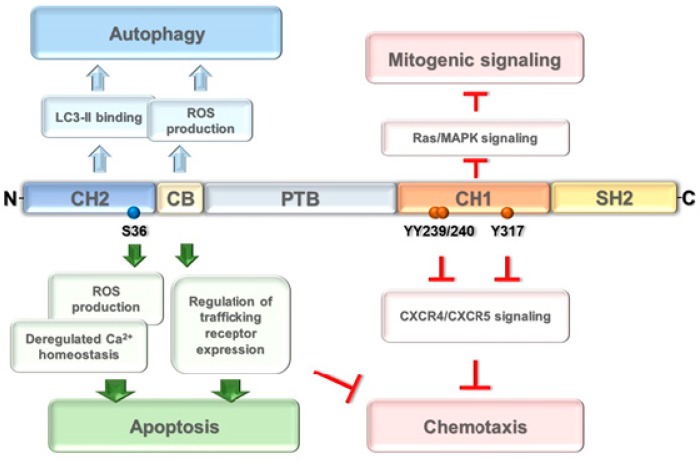
Structure and function of p66Shc. The N-terminal CH2 and cytochrome c binding (CB) domains promote apoptosis through reactive oxygen species (ROS) production, deregulation of Ca^2+^ homeostasis and by modulating the expression of trafficking receptors. They also specify p66Shc-mediated autophagic responses through LC3-II binding. The central CH1 domain, through phosphorylation of the three tyrosines 239/240 and 317, is responsible for the negative regulation both of CXCR4/CXCR5-dependent chemotaxis and of Ras/MAPK-dependent mitogenic signals.

The Shc family of adaptor proteins includes four members—ShcA, B, C, D—that can be expressed as multiple isoforms, dependent on the cell type. All Shc proteins share a unique highly conserved domain organization which represents the family signature. This consists of a N-terminal phosphotyrosine-binding (PTB) domain, a central proline-rich collagen homology (CH1) domain containing phosphorylatable tyrosine residues that are docking sites for SH2 domain-bearing proteins and a C-terminal SH2 domain [15] (Figure 1). 

ShcA is expressed as three isoforms of 66, 52 and 46 kDa, respectively, which share the PTB-CH1-SH2 modular structure, with three phosphorylatable tyrosines residues at positions 239/249 and 317 in the CH1 domain. p66Shc has an additional N-terminal CH domain, termed CH2, containing a phosphorylatable serine residue at position 36 [15] (Figure 1). Interestingly, the three ShcA isoforms differ remarkably in expression, subcellular localization and function. The major isoforms, p52Shc and p46Shc, are constitutively co-expressed from the same gene promoter but have different subcellular localizations. Initially identified as an adaptor that couples receptor tyrosine kinases to the Ras/MAP kinase pathway [16], p52Shc is a cytosolic protein that has been implicated in mitogenic, survival and chemotactic signaling by a large variety of surface receptors [15]. While only differing from p52Shc by the lack of the most N-terminal amino acid stretch, p46Shc localizes to mitochondria, where its function remains unknown [17]. At variance with the two shorter isoforms, p66Shc is expressed from an alternative gene promoter which is controlled both epigenetically and transcriptionally and is responsive to extracellular stimuli [18]. Intriguingly, the additional CH2 domain endows p66Shc with opposite functions compared to p52Shc. In the cytosol, p66Shc acts as a competitive inhibitor of p52Shc by preventing its recruitment to activated tyrosine kinase receptors and sequestering Grb2 [19,20]. This function requires S36 phosphorylation in the CH2 domain [20,21]. p66Shc also has pro-oxidant activities that are independent of its adaptor function. S36-phosphorylated p66Shc regulates the subcellular localization of the transcription factor FOXO1, which controls the expression of reactive oxygen species (ROS)-scavenging enzymes, leading to an elevation of the levels of intracellular ROS [22]. Interestingly, p66Shc also acts as a pro-oxidant enzyme. This activity is carried out by a pool of p66Shc localized in the mitochondrial intermembrane space, which increases under conditions of cellular stress. There it binds to and oxidizes cytochrome c, thereby interrupting the respiratory chain, which results in ROS production and increased susceptibility to apoptosis [23]. The mitochondrial localization depends on S36 phosphorylation and on the PIN1 isomerase, which acts as a chaperone [24], while the pro-oxidant function maps to a cytochrome c binding site at the C-terminus of the CH2 domain [23] (Figure 1). The adaptor and pro-oxidant functions of p66Shc make this molecule an important hub in the regulation of the pathways triggered by tyrosine kinase receptors and in the cellular response to stress.

### 2.2. Functions of P66Shc in Lymphocytes

#### 2.2.1. P66Shc Negatively Regulates Antigen Receptor Signaling

Similar to other tyrosine kinase-coupled receptors, p52Shc and p66Shc play opposite roles in signaling by antigen receptors. In T lymphocytes, p52Shc is recruited to the T cell receptor (TCR) following ligand binding and becomes phosphorylated on YY239/240 and Y317 in the CH1 domain by the ζ chain associated protein kinase 70 (ZAP-70) and Lck, resulting in the generation of two docking sites for SH2 domain-containing proteins [25,26,27]. p52Shc phosphorylation promotes its interaction with the Grb2:Sos complex, which in turn results in the activation of Ras and initiation of the MAPK cascade [25]. Similar to p52Shc, p66Shc is recruited to the activated TCR and becomes phosphorylated on the same CH1 tyrosine residues, which results in the inhibition of the Ras/MAPK pathway [21,28]. p66Shc exerts its inhibitory activity on TCR signaling by competing with p52Shc for the interaction with ZAP-70, both in basal conditions and following TCR engagement. This results in the defective recruitment of p52Shc to the activated TCR [21], supporting a role for p66Shc as a negative regulator of the mitogenic cascade triggered by the TCR.

In B lymphocytes the mechanisms of p52Shc recruitment to the B cell receptor (BCR) and the activation of the downstream pathway closely resemble the ones described for the TCR. BCR engagement results in p52Shc phosphorylation on YY239/240 and Y317 by the kinases Lyn and SYK [29], allowing Grb2:Sos recruitment to the membrane and Ras/MAPKs activation [30,31]. Similar to what occurs in TCR signaling, p66Shc acts as antagonist of the BCR-triggered mitogenic signaling. Inhibition of BCR signaling by p66Shc occurs at an early step in the cascade, as indicated by the observation that the BCR-dependent phosphorylation of the key initiating protein tyrosine kinase, SYK, was impaired in the presence of p66Shc [13].

The role of p66Shc as a negative regulator of antigen receptor signaling has been confirmed in vivo. T and B lymphocytes from p66Shc mice display enhanced proliferation in response to antigen receptor engagement in vitro and more robust immune responses both to vaccination and to allergen sensitization in vivo [28], supporting the notion that p66Shc expression controls lymphocyte activation and ligand-dependent responsiveness. It should be underscored that p52Shc participates in signaling by other immunoreceptors, including Fc receptors in B cells and mast cells, and NK receptors in natural killer cells [32,33,34,35]. This suggests that p66Shc has the potential to tune down both adaptive and innate immune responses. In support of this notion we reported that p66Shc inhibits FcεR signaling and mast cell degranulation both in vitro and in vivo [28,36,37].

#### 2.2.2. P66Shc Negatively Regulates Lymphocyte Chemotaxis

Antigen receptor ligation has been reported to induce signals leading to the internalization of CXCR4 in both T cells [38] and B cells [39,40]. In turn, CXCR4 requires surface expression of the antigen receptors to signal, as documented in T cells [41] and B cells [42]. These findings, taken together with the fact that antigen and chemokine receptors share many signaling mediators [43] [44,45], suggest a mechanism of transactivation by chemokine receptors. This has been demonstrated for CXCR4 which physically associates with the TCR on binding its cognate chemokine, leading to ITAM phosphorylation in the CD3ζ chain and downstream signaling [41,46]. We have identified p52Shc a crucial player in TCR transactivation by CXCR4 that participates as an early component in the CXCR4 signaling cascade to control T cell migration as well as ligand-dependent CXCR4 internalization [41]. Interestingly, p66Shc acts as a negative regulator of the chemotactic responses triggered by both CXCR4 and CXCR5 in B cells [46]. We found that p66Shc exerts this function by interacting with the chemokine receptors and promoting the assembly of an inhibitory complex that includes the tyrosine phosphatase SHP-1 and the lipid phosphatase SHIP-1. This results in impaired phosphorylation of the tyrosine kinases SYK and BTK and of the guanine exchanger Vav [47], and in defective chemokine-dependent [Ca^2+^]_i_ mobilization [48], a function that we mapped to the phosphorylatable tyrosine residues in the CH1 domain of p66Shc [46]. Hence, by interfering with the reorganization of the actin cytoskeleton triggered by CXCR4 and CXCR5, tyrosine-phosphorylated p66Shc controls B-cell chemotaxis [46].

#### 2.2.3. P66Shc Promotes Lymphocyte Apoptosis

The role of p66Shc as a mediator of apoptotic responses to oxidative stress, initially characterized in fibroblasts [49], is conserved in T lymphocytes, where p66Shc expression results in increased susceptibility to apoptosis induced by both pharmacological and physiological stimuli [21,50]. The pro-apoptotic activity of p66Shc was mapped to the phosphorylatable S36 residue in the CH2 domain [21]. p66Shc exerts its pro-apoptotic activity by impairing mitochondrial integrity, with associated dissipation of the mitochondrial transmembrane potential and cytochrome c release [50], an activity which requires both Lck and CamKII [51]. The pro-apoptotic activity of p66Shc was further associated to its ability to modulate the balance of pro-apoptotic and anti-apoptotic members of the BCL2 family (BCL2L1, BAX) towards the pro-apoptotic ones by affecting transcription of the respective genes [21]. Moreover, p66Shc expression affects T cell survival by deregulating Ca^2+^ homeostasis, as observed in p66Shc-overexpressing T cells which show a defect in their Ca^2+^-handling ability under conditions of Ca^2+^ overload [50]. The pro-apoptotic activity of p66Shc is conserved in B cells, as demonstrated by its ability to enhance B-cell apoptosis induced by prolonged surface Ig cross-linking [13] or by treatment with the pro-apoptotic drug fludarabine [52]. Similar to T cells, this function involves a modulation of the expression of the BCL2 family members, with a reduction in the levels of anti-apoptotic members (BCL2, BCL2L1) and an increase in the levels of pro-apoptotic members (BAX, BAK). Taken together with the decrease in BCR-triggered, AKT-dependent survival signaling observed in the presence of p66Shc [13] the data collectively highlight a role p66Shc as an inhibitor of B-cell survival.

Interestingly, we have recently implicated p66Shc in selective B cell autophagy/mitophagy. p66Shc participates in two distinct, yet functionally intertwined steps in this process. First, interruption of the mitochondrial respiratory chain by p66Shc results in a decrease in ATP production, which leads to the activation of the autophagy-promoting kinase AMPK [53,54]. Second, ROS production by p66Shc at mitochondria disrupts their integrity, which results in protein ubiquitylation at the outer mitochondrial membrane and recruitment of AMPK, thereby promoting local phagophore assembly. These functions require the ROS-elevating activity of p66Shc mediated by cytochrome c binding. Full execution of autophagy also requires the presence of an LC3-II binding motif that we mapped to the CH2 domain [54] (Figure 1). These results place p66Shc at the cross roads of multiple pathways that control cell survival through its ability to sense and respond to survival signaling, metabolic status or cellular stress [55].

#### 2.2.4. The Pathogenic Effects of Altered P66Shc Expression

As mentioned above, at variance with the two other ShcA isoforms, p66Shc expression is tissue-restricted and controlled by an alternative promoter [18]. Lymphocytes express lower levels of p66Shc compared to the other two isoforms, a feature that in T lymphocytes has been associated with hypermethylation of the p66Shc promoter [56]. The implication of DNA methylation as a major mechanism of epigenetic control of p66Shc transcription underscores the importance of a tight regulation of its expression.

A dysregulation of the expression levels of p66Shc has been associated with the development of several diseases. Abnormally high p66Shc expression has been recently implicated in liver fibrosis, where it mediates mitochondrial ROS production and tissue damage [57]. Moreover, overexpression of p66Shc promotes the development of Alzheimer’s disease by enhancing neuronal cell sensitivity to the pathogenic peptide amyloid beta [58]. A causative role for enhanced p66Shc expression and ROS production in ischemic brain injury and cardiovascular disease has been also suggested [59,60], although further studies are needed to better clarify this issue.

Abnormalities in p66Shc expression have also been causally linked to immune disorders. Mice lacking p66Shc expression develop an age-related systemic lupus-like autoimmune disease characterized by spontaneous peripheral T- and B-cell activation and proliferation, autoantibody production and immune complex deposition in both skin and kidney. This results in autoimmune glomerulonephritis and alopecia, the latter of which is associated with spontaneous mast cell degranulation [28]. Related to the topic of this review, p66Shc expression is profoundly impaired in leukemic cells from CLL patients compared to healthy donors [13], underscoring the importance of its regulation in disease control.

## 3. P66Shc Deficiency in CLL B Cells Contributes to Disease Pathogenesis

CLL cells resemble phenotypically [61] as well as genetically [62] mature, antigen-experienced CD27^+^ memory B cells. These cells are unable to mature into immunoglobulin-producing plasma cells and therefore cannot respond to infections, which is the principal cause of mortality among CLL patients [63,64]. A heterogeneous clinical course characterizes CLL. While a subgroup of patients has a benign disease that can remain stable for several years without the need of any therapy, other patients develop an aggressive disease form which requires early treatment. A plethora of prognostic factors has been identified to individually define the risk for disease progression and develop personalized therapeutic approaches. In addition to the widely used Rai-Binet clinical staging systems [65], biological markers have been identified which include overexpression of CD38 [66] and ectopic expression of ZAP-70 [67]. Genetic parameters hold a prominent role in CLL prognostication. Unmutated immunoglobulin heavy chain variable region (IGHV) genes are usually associated to the most aggressive disease presentation (U-CLL patients), while mutated IGHV genes are associated to mild disease (M-CLL patients) [68]. Furthermore, 13q deletions, chromosome 12 trisomy, 11q deletions and 17p deletions are the cytogenetic aberrations most frequently found in CLL patients [65].

In 2010, Capitani and colleagues found that CLL cells have a profound defect in the expression of p66Shc, with the lowest residual levels in leukemic cells from patients with unmutated IGHV genes [13], a marker of unfavorable prognosis [68]. This observation was recapitulated in the Eµ-TCL1 mouse model of human CLL [69] where we found p66Shc expression to decline during disease progression until its almost complete loss in mice with overt leukemia [52]. *p66Shc* deletion in Eµ-TCL1 mice through a genetic approach resulted in accelerated leukemogenesis and enhanced disease aggressiveness, which was associated with prolonged survival and chemoresistance of the p66Shc-deficient leukemic cells [52]. The fact that p66Shc expression levels correlate with poor CLL prognosis and severity [13,52] suggests that the p66Shc expression defect in CLL cells may contribute to their aberrant biological behavior.

The heterogeneous clinical behavior of CLL and the expanding number of prognostic and predictive markers make disease management extremely hard. To date, the key decision making biomarkers in CLL are *TP53* defects—deletion of the 17p13 locus and/or mutation(s) within the *TP53* gene—which are associated with resistance to chemoimmunotherapy and poor clinical outcome [5,70,71]. Surprisingly, no correlation between p66Shc expression and *TP53* defects has been observed in leukemic cells from either CLL patients or Eµ-TCL1/p66Shc^−/−^ mice [52], notwithstanding a documented crosstalk between p53 and the ROS-elevating activity of p66Shc [49]. However, molecules other than p53, which contribute to control the death/survival balance, have been found to be altered as a consequence of the p66Shc expression defect in CLL B cells, both at the transcriptional and post-transcriptional level. Here we describe the mechanisms whereby p66Shc deficiency helps leukemic cells to escape apoptosis by regulating these molecules.

### 3.1. P66Shc Deficiency Modulates Gene Transcription to Prolong CLL B Cell Survival

#### 3.1.1. P66Shc Expression Impinges on the BCL2 Family Balance

The ratio of pro- versus anti-apoptotic members of the BCL2 family of apoptosis-regulating proteins has been found to be altered in a number of diseases [72]. These include CLL, where it contributes to the shift of leukemic cells towards survival and correlates with chemoresistance and poor prognosis [73,74]. Both BCL2 and MCL1 are overexpressed in CLL cells, where they mediate tumor cell survival and have been associated with resistance to therapy [74,75]. Less than 50% of CLL patients have deletion, alteration or downregulation of mir-15a and mir-16-1 [76] in the *DLEU2-mir-15-16* cluster, which both target BCL2 [77] and lead to enhanced BCL2 expression and increased resistance of leukemic cells to apoptosis [77]. Pro-apoptotic components of the same family, namely BAX and BAK, are concomitantly less expressed [13,74], making this protein family an attractive therapeutic target for the treatment of CLL [78]. The results of recent clinical trials with the BH3-mimetic drug Venetoclax [79], which potently induces apoptosis in CLL cells [80], support the potential of this approach.

**Figure 2:**
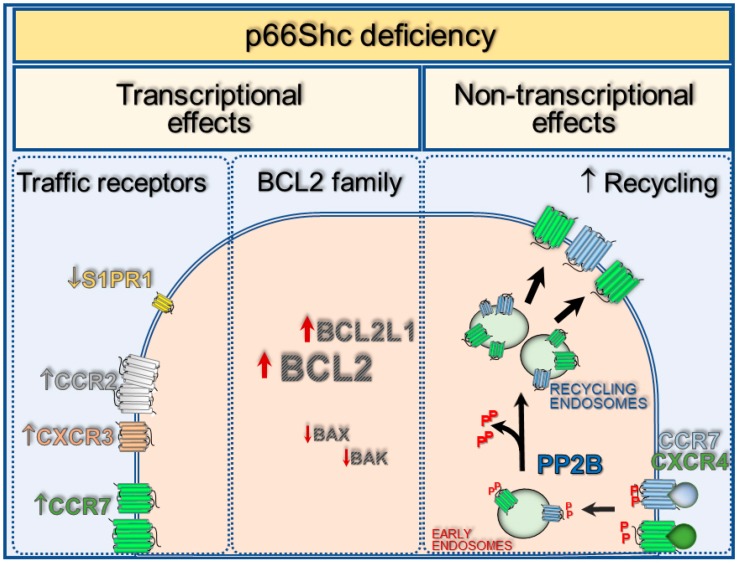
Transcriptional and non-transcriptional effects of p66Shc deficiency in CLL B cells. p66Shc deficiency in CLL B cells impinges on the transcription of genes encoding trafficking receptors, which enhances the expression of the homing receptors CCR2, CCR7 and CXCR3, while lowering the expression the egress receptor sphingosine-1-phosphate receptor 1 (S1PR1). p66Shc also modulates the balance of BCL2 family members by promoting the expression of the pro-survival BCL2 and BCL2L1 while lowering the pro-apoptotic BAX and BAK. By enhancing Ca^2+^ mobilization, p66Shc deficiency translates to enhanced serine-phosphatase activity of PP2B/Calcineurin on the endosomal pool of the homing receptors CCR7 and CXCR4, thereby enhancing their recycling back to the plasma membrane to replenish the complement of signaling-competent receptors.

p66Shc deficiency impinges on the BCL2 family balance (Figure 2), shifting the ratio of BCL2 family members towards the pro-survival BCL2/BCL2L1, to the detriment of the pro-apoptotic BAK/BAX in the p66Shc-deficient CLL B cells [13] as well as in leukemic cells from Eµ-TCL1 mice [52]. p66Shc reconstitution by transient transfection in CLL B cells normalizes the BCL2 family ratio [13], providing proof-of-concept that p66Shc is implicated in the transcriptional regulation of the BCL2 family.

#### 3.1.2. P66Shc Expression Impinges on the Expression of Trafficking Receptors

CLL B cells are characterized by trafficking abnormalities that strongly contribute to their prolonged survival. Their enhanced ability to home to peripheral lymphoid organs and bone marrow, together with their failure to efficiency egress therefrom, leads to a prolonged residency into the lymphoid stromal niche, where they acquire proliferation and survival cues [10,12]. The altered traffic of CLL B cells can be ascribed, at least in part, to abnormally high levels of chemokine receptors at their surface [12,52,81,82,83,84,85]. CLL B cells overexpress chemokine receptors which guide lymphocyte homing to both nodal and extranodal sites, including CXCR4, CCR7, CXCR5, CXCR3 and CCR2 [52,81,82,83,84,86,87,88,89,90,91,92,93]. Clinical correlation of poor prognostic CLL markers (unmutated IGHV genes, infiltration of nodal and extranodal organs) with high expression levels of these homing receptors has been reported [52]. Importantly, enhanced expression of homing receptors inversely correlates with the residual p66Shc expression levels of CLL B cells [52,84].

On the other hand, p66Shc deficiency in CLL B cells from patients with unfavorable prognosis directly correlates to their abnormally low levels of the surface receptor S1PR1 [84] whose ligand, the sphingolipid S1P (sphingosine 1-phosphate), promotes lymphocyte egress from lymphoid organs [94]. The enhanced expression of homing receptors, and the defective S1PR1 expression harbored by CLL B cells from these patients, both contribute to the accumulation of CLL cells in the pro-survival niche of lymphoid organs [12]. Importantly, forced p66Shc expression in CLL cells normalizes the expression of these homing and egress receptors and restores chemotaxis towards the respective ligands [52,84], implicating residual p66Shc expression levels as a checkpoint of infiltrating vs non-infiltrating leukemia.

#### 3.1.3. The Ability of P66Shc to Modulate Gene Transcription Is Dependent on Its Pro-Oxidant Activity

The involvement of the cellular redox status in cancer onset and development is controversial [95]. Nevertheless, prolonged oxidative stress resulting from the imbalance between production and detoxification of ROS has been observed in several tumors which in turn develop peculiar anti-oxidant strategies to overcome ROS-related cell death [95]. Drugs aimed to control the oxidative status of tumor cells might therefore provide interesting innovative therapeutic approaches.

Several transcription factors are sensitive to the redox status of the cell and hence participate in the cellular defenses against oxidative stress. These include the transcription factor nuclear factor erythroid 2-related factor 2, which regulates the expression of detoxifying and anti-oxidant enzymes and anti-apoptotic proteins [96]; the transcription factor NF-κB, whose activation via the canonical and non-canonical pathways is either positively or negatively modulated by ROS at multiple levels [97]; and the FOXO family of transcription factors, whose activity is strongly sensitive to the intracellular ROS content [98].

As the result of its ROS-elevating activity (see Section 2.2.3), p66Shc has the potential to impact cellular redox status. We recently reported that the intracellular ROS content is reduced in CLL B cells when compared to healthy B cells [52]. Furthermore, we showed that the pro-oxidant activity of p66Shc impinges on its ability to indirectly modulate gene transcription [52,84]. Transcription of *ccr7*, *ccr2*, *cxcr3* and *s1pr1* is indeed either enhanced or repressed by the expression of a p66Shc mutant lacking the pro-oxidant activity [52,84], supporting the notion that neoplastic B cell residency in both lymphoid and non-lymphoid niches [52] depends on the ROS-elevating activity of p66Shc. Putative ROS-sensitive transcription factors, specifically modulated in CLL B cells by the p66Shc defect, have not yet been identified. However, previous data demonstrating that the activity of FOXO1 is upregulated in p66Shc^−/−^ mouse embryonic fibroblasts [22] strongly corroborate the hypothesis that p66Shc deficiency in CLL B cells can affect gene transcription as the result of a decrease in intracellular ROS. Of note, other reports demonstrate that intracellular ROS are enhanced in CLL B cells compared to normal B cells due to increased mitochondrial respiration rate [99], suggesting that further studies are needed to clarify this issue.

### 3.2. Receptor Recycling as an Alternative Mechanism for B Cell Trafficking Regulation by P66Shc

De novo gene transcription enhances protein availability through prolonged and expensive molecular processes. In some instances, however, alternative low-cost mechanisms are exploited which, by saving both energy and nutrients, contribute to enhance the net protein amount. Among these mechanisms, recycling, the fast receptor re-exposure at the cell surface after ligand-dependent internalization, is exploited by chemokine receptors to modulate their own surface levels and their migratory response [12,100]. Lymphocytes exploit downmodulation and recycling of chemokine and S1P receptors in the absence of de novo gene expression to rapidly modify their surface levels, and this ensures their cyclic traffic through the peripheral lymphoid organs to detect the potential presence of pathogens and receive survival signals [12].

Cancer cells take advantage of low-cost mechanisms and, in some instances, subvert them to efficiently compete for environmental resources. We recently reported that CLL cells, especially those from patients with aggressive disease, enhance recycling of the homing receptors CCR7 and CXCR4 from the intracellular pool to the plasma membrane to increase their surface levels [92]. This enhancement can be ascribed, at least in part, to the enhanced dephosphorylating activity of the Ca^2+^-dependent phosphoserine phosphatase PP2B, also known as Calcineurin [101], which promotes transit of both CXCR4 and CCR7 from the Rab5^+^ (early endosome) to the Rab11^+^ (recycling endosome) endosomal compartment and then to the cell surface [48]. PP2B activation was found to be a direct consequence of the enhanced ability of CXCR4 and CXCR5 to trigger Ca^2+^ mobilization in CLL B cells upon ligand binding [48].

We demonstrated that the p66Shc expression defect harbored by CLL B cells is implicated in their enhanced recycling of CXCR4 and CCR7 [48]. Consistent with the finding that, by attenuating Ca^2+^ mobilization, p66Shc interferes with the signaling pathways elicited by ligand-stimulated chemokine receptors [46], the enhancement in the dephosphorylating activity of PP2B towards serine-phosphorylated endosomal receptors in CLL B cells could be reversed by forced p66Shc expression. This would result in normalization of CXCR4 and CCR7 recycling to the plasma membrane and B cell migration in response to the respective chemokine ligands [48] (Figure 2). Of note, while the enhanced expression of CXCR4 only relies on potentiated recycling, with no evidence of increased transcription, CCR7 overexpression in CLL cells relies both on enhanced recycling and enhanced gene transcription. Together, these mechanisms contribute to strongly amplify the surface levels of this receptor to promote leukemic cell homing to the lymphoid niche.

## 4. P66Shc Reconstitution as a Future Perspective for the Treatment of CLL

Current evidence highlights the multifaceted role of p66Shc in B cells and the profound consequences of its deficiency on B cell apoptosis, survival and chemotaxis, all of which are dysregulated in CLL B cells. Due to its ability to participate as a negative regulator in multiple signaling pathways, its role may not be limited to these processes. For example, it can be hypothesized the enhanced BCR-triggered signaling of CLL B cells with unmutated IGHV genes [102], which have the lowest levels of residual p66Shc [13], may be related at least in part to their p66Shc expression defect.

Several pieces of evidence indicate that p66Shc deficiency is implicated in the abnormal behavior of CLL cells, thereby participating in CLL pathogenesis. As mentioned above, p66Shc expression declines along with disease progression in leukemic cells form Eµ-TCL1 mice which, similar to human CLL cells, express abnormally low levels of p66Shc [52]. Moreover, leukemia presentation is more severe in Eµ-TCL1/p66Shc^−/−^ mice [52]. Importantly, p66Shc expression inversely correlates with CLL B cell chemoresistance [52]. Restoration of the normal p66Shc levels therefore acquires therapeutic relevance for the treatment of CLL. The potential of this approach is exemplified by Malignant Melanoma, a solid tumor characterized by defects in the apoptosis machinery, whose treatment with the anti-tumoral drug Gambogic acid enhances p66Shc expression, intracellular ROS content and BAX-mediated apoptosis, thereby inhibiting tumor cell proliferation [103]. At variance with molecules currently targeted by pharmacological treatments (such as BCL2 or BTK) whose levels are changed or whose activation is affected only in a limited proportion of patients, the defect in p66Shc expression is a feature shared by leukemic cells from CLL patients both with mild and aggressive presentation. This in turn makes p66Shc reconstitution an attractive therapeutic target. We showed that the abnormalities harbored by CLL B cells can be reversed by forcing p66Shc expression in CLL B cells in vitro [48,84], further supporting the notion that strategies aimed to normalize p66Shc are promising candidates to treat this disease and overcome leukemic cell chemoresistance.

P66Shc transcription is controlled by promoter methylation and histone deacetylation in both primary and transformed cells, including T cells [18,56]. Together with the fact that the general pattern of DNA methylation increases in Eµ-TCL1 mice during disease development [104], these observations suggest the possibility that this epigenetic mechanism may regulate *p66shc* transcription in CLL B cells. However, p66Shc expression is not controlled by promoter methylation in B cells [13], indicating that other mechanisms are responsible for p66Shc silencing. Interestingly, histone acetylation has been recently reported to enhance p66Shc transcription in human retinal endothelial cells subjected to high glucose as a pro-oxidant stimulus [105], opening new scenarios in the complex regulatory mechanisms underlying p66Shc expression.

We recently found that p66Shc is regulated by the transcription factor STAT4, a crucial mediator of the IL-12-triggered signaling pathway controlling differentiation of T helper (Th) 1 cells and their inflammatory responses [106]. We showed that STAT4 expression is profoundly impaired in CLL B cells [107]. Interestingly, by triggering phosphorylation and activation of STAT4, IL-12 coordinately increases STAT4 and p66Shc expression and enhances B cell apoptosis [107]. This suggests the possibility that STAT4 agonists able to stimulate the residual STAT4 in CLL B cells could normalize p66Shc expression. p66Shc can be restored both in CLL B cells [48] and in leukemic cells from Eμ-TCL1 mice [52] by treatment with the BTK inhibitor ibrutinib, which also promotes STAT4 expression in leukemic cells [52]. Of note, patients treated with ibrutinib experience a massive mobilization of leukemic cells from lymphoid organs to peripheral blood [108], which is at least in part related to the normalization of p66Shc and, consequently, of S1PR1 expression [48,84]. It is noteworthy that ibrutinib does not specifically target the STAT4/p66Shc pathway, which seems rather a side effect of this drug, and a compound that selectively targets this pathway remains to be identified.

## 5. Conclusions

The interplay of CLL B cells with the stromal microenvironment has emerged as a key factor in disease pathogenesis. Not surprisingly, homing of leukemic cells to the protective stromal niche of lymphoid organs and their accumulation therein has recently attracted major attention [109]. Notably, nodal and extranodal infiltration, a feature of CLL patients with aggressive disease presentation, is associated the lowest levels of residual p66Shc [13,52]. Hence, the profound consequences of p66Shc deficiency on the expression and function of trafficking receptors and on the resulting outcome on disease presentation warrant further studies to fully elucidate the mechanisms linking p66Shc deficiency to disease.

It is worth noting that the stromal microenvironment fosters survival and chemoresistance of leukemic cells both in a cell-dependent and cell-independent manner [3], highlighting the mechanisms that control leukemic cell trafficking and their molecular regulators as important areas of investigation. Soluble factors secreted by cancer cells, such as IL-6, have been recently found to acquire pro-tumoral functions by stimulating the pro-survival activities of stromal cells [110], thereby contributing to cancer progression. Whether and how the p66Shc defect on CLL B cells impacts their ability to shape the leukemic microenvironment is still unknown, but definitely deserves to be investigated.
